# Navigating the Treacherous Waters of Geriatric Complexity and Heterogeneity With the Help of Team Science

**DOI:** 10.1093/geroni/igaa057.2911

**Published:** 2020-12-16

**Authors:** George Kuchel

**Affiliations:** University of Connecticut, Farmington, Connecticut, United States

## Abstract

Multifactorial complexity and heterogeneity challenge the care of older adults and research into the pathophysiology of common geriatric syndromes. Multicomponent interventions matching intervention components with individual risk factors are grounded in precision medicine by ensuring that interventions may be offered to those who will more likely beneﬁt, sparing expense and side effects for those who will not. Nonetheless, the development of mechanism-guided interventions has been hampered by failure to identify single mechanisms for effective targeting within this multifactorial complexity, a problem worsened by historical barriers between research disciplines and silos. Geroscience-guided interventions target biological hallmarks of aging representing mechanisms that geriatric syndromes share with aging. We will present examples of multidisciplinary bench-to-bedside translational science seeking to transform the care of common geriatric conditions as diverse as frailty, voiding disorders and immunization against influenza and pneumococcal infections via geroscience-guided therapies applied with a greater emphasis on heterogeneity of aging and targeting.

